# Editorial: Robotics, Autonomous Systems and AI for Nonurgent/Nonemergent Healthcare Delivery During and After the COVID-19 Pandemic

**DOI:** 10.3389/frobt.2022.886926

**Published:** 2022-06-08

**Authors:** A. L. Trejos, S. F. Atashzar, S. P. DiMaio, P. M. Pilarski, M. Tavakoli

**Affiliations:** ^1^ Western University, London, ON, Canada; ^2^ New York University, New York City, NY, United States; ^3^ Intuitive Surgical, Inc., Sunnyvale, CA, United States; ^4^ University of Alberta, Edmonton, AB, Canada

**Keywords:** robotics, autonomous systems, artificial intelligence, healthcare, COVID-19

In an emergency, all resources are dedicated to ensuring survival. This was also true of the COVID-19 pandemic, in which, in an effort to keep people safe, research was directed to understanding the virus better and ensuring vaccines were developed. However, the vulnerability of our health care system was also exposed when we saw nonurgent and nonemergent cases fall through the cracks while resources were directed at addressing the emerging health crisis. This special issue aimed to capture novel research directions and perspectives on technological advances that can be put in place to support health care: from novel techniques for diagnosing and treating COVID-19 and preventing spread, to tools and procedures that support other aspects of our health during and after the pandemic. A total of 33 articles by 180 leading authors were accepted from around the world, demonstrating a significant effort from the robotics and artificial intelligence (AI) communities to help reduce the impact of the pandemic on our overall health. The articles have been divided into general themes, as described below.

## Prevention of Community Spread

The first line of defense in a pandemic is to prevent the spread of the virus within the community, and a large component of prevention is proper cleaning and disinfection. Towards this end, the paper by Nasirian et al. proposes an end-to-end coverage path planning (CPP) method using a novel graph representation of the environment that can generate a continuous and uninterrupted collision-free path for an autonomous mobile robot. The proposed method is able to generate an optimal path that can reduce the disinfection task completion time and cost through shorter travel distances and a smaller number of turns than other approaches.

Complementary to disinfection are other ways to prevent the spread of the virus. The work by Michelin et al. aimed to prevent people from touching their faces. A convolutional neural network (CNN) algorithm using data from an inertial measurement unit (IMU) at the wrist was able to predict when a person was about to touch their face. The system then provided sensory feedback in various forms, with vibrotactile feedback providing the fastest response, the best success rate, and the best user experience. Vicente et al. examine robotics and automation as an approach to reducing the transmissibility of COVID-19 in healthcare environments. Through modeling of temporal and spatial factors in geriatric units, they present a view into how robotic integration at key points in the care environment can have a large potential impact on the spread of pathogens in these units. To demonstrate the breadth of potential applications for robots to prevent viral spread, a review paper by Onaizah et al. evaluated the needs and challenges of designing robotic flexible endoscopes during a pandemic. By making a few minor adjustments to existing platforms, or by considering platforms in development, authors argue that significant benefits can be gained during infection control scenarios.

## Diagnostic Technologies

Once the virus has spread, the second line of defense is to minimize exposure of healthy individuals by identifying and isolating those who are infected. For this purpose, a significant effort has been dedicated by the research community to identify better ways of diagnosing COVID-19 and the secondary effects that result from it. Given the potential health hazards during traditional swab sampling, a sensorized, self-administered oral swab is designed and fabricated by Kumar et al. using a closed-loop kinematic chain and a kirigami-based deployable telescopic tubular structure. Compared to traditional swabbing procedures and robot-assisted swab system, the proposed oral swab is simpler, less resource-intensive, and more convenient for self-administration. Also for swab sampling, Li et al. propose a flexible transoral robot in a teleoperated configuration and with a flexible mechanism. The proposed transoral robot realizes dexterous sampling and a tongue depressor is used to prevent the tongue’s interference during the sampling. In an experiment with a human phantom, the usability of the robot is demonstrated. On the imaging side, the article by Deng and Li provides a comprehensive review of published work related to the use of AI and Deep Learning methods for segmentation, detection, diagnosis, and severity assessment for COVID-19 from X-RAY and computed tomography (CT) chest images. Ninety-six published studies are reviewed, with a discussion of future directions and thoughts on data fusion strategies for integrating multi-modality image data for COVID-19 examination. In order to better allocate clinical resources through early diagnosis, Heidarian et al. propose the use of capsule-network-based deep learning algorithms for interpreting CT data with increased spatial acuity, thanks in part to their use of capsule networks in place of more standard convolutional architectures. Their COVID-FACT framework was shown to require less manual intervention by domain experts while achieving accuracy, sensitivity, and specificity suitable for COVID-19 diagnosis. Similarly, in Tse et al., the authors survey the use of CT and its enhancements through methods from the field of artificial intelligence as an approach to COVID-19 diagnosis. They suggest further ways that the automation of CT through advanced computing technology can help combine with other existing approaches to COVID-19 assessment and mitigate risk factors in the supply and deployment of other gold-standard diagnostic approaches. Finally, McDermott et al. examine how lung ultrasound (LUS) is being used to diagnose COVID-19 and note difficulties in interpreting LUS by non-specialized operators. The authors survey algorithms that could potentially be used to automate the task of disease detection, severity classification, and patient triage. A comprehensive and critical review of image processing for lung ultrasound in the context of COVID-19 screening and diagnosis is presented.

## COVID-19 Patient Care

Once people are infected with the COVID-19 virus, treatment focuses on symptom management while the body heals; however, in many cases, patients have become severely ill with respiratory tract infections. When the respiratory illness is severe, some COVID-19 patients have required mechanical ventilation to support respiration while healing. Three papers in this special issue were related to the use of mechanical respirators. Mitros et al. presented the design of a 7-DOF robotic bronchoscope that allows accurate sampling of the distal lung in critical care mechanically ventilated patients, in order to improve diagnosis and treatment of lung disease. A prototype of a continuum robot is presented, as well as its mathematical model demonstrating high accuracy. In another paper by Xiao et al. a flexible trans-oral mini-robotic system incorporating a robotic needling technology is proposed to accurately access the cervical trachea in mechanically ventilated patients. Using an “inside-out” approach to the initial trachea puncture, the proposed system can cause fewer complications on the patient’s neck and trachea. Finally, considering that the large ventilators that currently exist in intensive care units (ICU) cannot be controlled remotely, even for simple setting adjustments, the work by Vagvolgyi et al. reports on the development of a simple, low-cost telerobotic system to control ventilators using a small Cartesian robot. Engineering system tests and usability tests are reported as successful, and operation time was reduced from 271 to 109 s in a preliminary evaluation.

Other areas of patient care could also be supported by robotic technologies, although additional research is needed. Sierra Marín et al. present a systematic analysis of the perception of healthcare professionals regarding the benefits and uses of robotic systems at various levels in medicine. The paper discusses the design of a perception questionnaire to assess the acceptance and required education for using robots at scale, in order to fight the COVID-19 pandemic. The paper reports on the results obtained from a survey of 41 healthcare professionals, exposing a low level of knowledge about robots and their potential benefits. In addition, the survey highlights that the concern of “being replaced by robots” remains in the medical community. However, overall enthusiasm was reported regarding the use of robots for a crisis such as a pandemic.

## Training of Medical Professionals

A large impact of the pandemic was felt in areas of healthcare that were not directly related to COVID-19. For example, teaching and education in healthcare was another area that suffered significantly as a result of closures and contact restrictions. For example, Motaharifar et al. focus on the use of virtual reality (VR), AI, and haptics for enhancing medical training during pandemics such as COVID-19 while reducing physical contact. This paper specifically focuses on how such technologies can help novice surgeons receive the needed medical training without the need for in-person attendance, using VR, AI, and haptics. It also provides a comprehensive survey describing recent technologies that can be used for reducing physical contact in medical institutes while keeping the quality of training at a high level. Similarly, a deep learning solution for encoding the movement behavior of expert surgeons to be used for rendering is presented in Fekri et al. The work focuses on the development of technologies for training novice residents in the orthopedic surgical drilling procedure, and thus augmenting their educational system through the development of a skill transfer system. The proposed system uses virtual fixtures and haptic guidance for novice surgeons in order to provide timely training for clinicians whose education may be affected by COVID-19. Cheng et al. identify serious challenges to dentistry education and advanced training due to limitations to in-person learning as a result of public health protective measures. As a possible solution, they introduce DenTeach—a remote dental education solution in portable suitcase format that allows dentistry schools to move practical training content to a remote delivery format. They illustrate the feasibility of DenTeach through case study analysis and key performance indicator assessment of hands-on learner interactions with their training device. Also related to dentistry education, Maddahi et al. explore ethical considerations in the use of simulation and robotics in healthcare education. The authors illustrate the principles of “roboethics” with a case study in self-guided dentistry education using such technologies. They examine challenges related to cognitive, affective, and psychomotor learning.

## Neuromuscular Rehabilitation

Regarding medical care, challenges created by the pandemic were particularly noticeable in neurorehabilitation, as the interruption of treatment resulted in critical problems, as highlighted by Lambercy et al. This paper presents the limitations in neurorehabilitation that occurred when the COVID-19 pandemic started and rehabilitation moved to a home setting, as well as some key directions to explore for providing neurorehabilitation from a distance. These include improvements in the usability of available technologies, the development of scalable rehabilitation technologies to account for the increasing number of patients, and clinically relevant and transparent AI to increase patients’ trust in the technologies. These ideas are supported by a review by Atashzar et al. on the utility of intelligent robotic solutions for isolated adults with neuro-musculoskeletal conditions. The authors argue that smart robotics and wearable technology can play an important role in the timely delivery of assistance and support to individuals when it is most needed in their care pathway. They further support the perspective that such approaches allow physical distancing and other protective measures suitable for use during an ongoing public health crisis, providing a sound argument for the use of automation in the care of more isolated end users. Similarly, a review paper by Manjunatha et al. focuses on in-home rehabilitation robotics as a medium to deliver the needed therapy during COVID-19, addressing an increasing concern related to the need for rehabilitation therapy delivery during the pandemic. Specifically, the paper focuses on the overloading of the rehabilitation facilities by post-intensive-care patients (after COVID-19 infection) who have developed neurological and physical symptoms and require a wide range of rehabilitative care. This paper conducts a literature review of various telerehabilitation frameworks and robotic solutions that can be used in a hybrid model for providing rehabilitation and assessment. The paper also provides insight regarding the social support and engagement of patients to further improve the benefits of telerehabilitation systems. Finally, a perspective article by Ahmed provides a brief review and the author’s opinion—as a neurologist—on how several routine protocols in healthcare may be improved using robotics and AI. The author indicates that such technologies can be merged with our home environments and various levels of healthcare delivery (from ambulance services to hospitalization and discharge) with a specific focus on how such technologies could potentially help the healthcare system during a health crisis such as the COVID-19 pandemic.

Various papers in this issue presented specific tools to aid neurorehabilitation. For example, arguing that early neurorehabilitation following a stroke cannot be delayed by the increased safety precautions resulting from the pandemic, Akbari et al. present a multi-agent framework for the development of intelligent rehabilitation systems for home use. They also provide a comprehensive review of existing devices that could be integrated into this framework for upper and lower-limb rehabilitation. The existing technologies for delivering mechanical tactile feedback (i.e., vibration, stretch, pressure, and mid-air stimulations) and those that can be integrated into home-based telerehabilitation practice are the special focus in a paper by Handelzalts et al. due to their low cost, compact size, and light weight. The advantages, opportunities, long-term challenges, and gaps with regard to practical implementations are discussed. Neurorehabilitation of gait using a social robot was presented in the work of Céspedes et al. In this study, the social robot monitored the patient’s posture and provided motivation and feedback, while quantitative and qualitative metrics were collected. The results show that significant progress was possible, as reflected in improved spinal posture, while the technology allowed physical distancing from caregivers.

Motivated by raising concerns regarding the negative effect of COVID-19 on immobilization and lack of access to physical therapy due to home confinements, isolation, or infections, Akbas and Mummolo focus on balance control as an indicator of corresponding movement disorders. In this work, a new computational framework is proposed, which can be used for the assessment of balance control in the homes of the users and patients. The authors discussed the application of their technique for home-care rehabilitation and assessment of balance exercises. In a case report by Carriere et al., the authors examine the opportunities that machine learning and machine intelligence methods, specifically natural language processing, present for delivering timely and appropriate acute and chronic rehabilitative care and assessment during an ongoing pandemic. As a concrete example, they discuss the deployment of a telehealth service called the Rehabilitation Advice Line (RAL) to address the immediate rehabilitation needs of patients. They then comment on how artificial intelligence and machine learning can be used to both enhance services like RAL, and leverage the data provided by such systems.

## Telemedicine and Remote Interactions

Other important methods and tools have been proposed to enable remote interactions, particularly to reduce the risk of virus transmission while maintaining patient care. Wazir et al. present a proof of concept for an emergency, remote monitoring and control system that can be used to retrofit dialysis machines with telerobotic manipulators for safely supporting the treatment of patients with acute kidney disease. The approach allows a human caregiver to remotely control a dialysis session using existing dialysis equipment, thus reducing the use of personal protective equipment (PPE) while minimizing the risk of COVID-19 exposure for healthcare staff and their patients. Grasse et al. investigate the use of a delivery robot for reducing disease exposure to high-risk residents and staff within a care facility. The system uses a voice interface to avoid hands-on interactions and investigates the effects of face masks on speech recognition quality and robustness. Meinhold et al. highlight the accelerated rate of interest in telemedicine and telehealth and focus on designing new technology, i.e., smart tendon hammer, which can be used to remotely conduct deep tendon reflex exams, which is a critical part of routing neurological assessments. The system is also able to differentiate correct and incorrect tapping locations with high accuracy, which is imperative as feedback for the user to provide a high-quality assessment. The proposed technology can allow novices to be also able to conduct the exam remotely, which can be critically useful during shutdowns of medical facilities and infrastructure due to pandemics such as COVID-19. Mehrdad et al. provide a perspective into the role that networked wearable technologies can play in remotely viewing and acting on the unfolding symptoms and outcomes of individuals during a continuing pandemic. They reinforce in their survey the idea that a networked wearable device (Internet of Medical Things—IoMT) can allow the acquisition of critical data for advanced monitoring and predictive disease mitigation, potentially enhancing policy making by governments and global health regions. Finally, Work by Feizi et al. showcases the potential of robotics and artificial intelligence for teleoperation in surgical interventions and training during ongoing COVID-19-related public health measures. They specifically examine intelligent robotics in the areas of robotics-assisted surgery, tele-examination pre- and post-surgery, and surgical training. As one important contribution of their perspective article, they illustrate the role that smart teleoperation can play in reducing the potential for virus transmission in a surgical setting.

Of important consideration during these trying times has been the need for a more human approach to care delivery. Lima et al. present the design, development, and testing of a multimodal robotic framework for a more affective human-robot interaction to support dementia patients using telemedicine. A hybrid face robot design that combines digital facial expressions with static 3D facial features is reported, and a contextual virtual assistant is introduced that enables the robot to adapt its facial expressions to the user’s speech in real time.

## Conclusion

Based on the contributions included in this special issue, a comprehensive picture has emerged on the state of the art of robotics and AI to address healthcare needs resulting from the pandemic, highlighting new and impactful directions that may be explored in future work. These research directions have aimed to address several COVID-19 related issues, including techniques for preventing the spread of the virus via cleaning, disinfection and shielding; diagnostic technologies that include swabbing and new image processing techniques; and methods for improving mechanical ventilation of critically ill patients. Research has also aimed to address issues not directly related to COVID-19, but that resulted from closures and long periods of quarantine required throughout the pandemic. These include technologies for teaching medical skills in various disciplines, new advances that aim to address rehabilitation of neuromuscular disorders, and other innovations in telemedicine. Although new technologies and techniques have been developed, further research and development is needed in order to fully exploit the benefits of the field for supporting the healthcare system now and in the years to come.

